# Intravaginal Practices, Bacterial Vaginosis, and HIV Infection in Women: Individual Participant Data Meta-analysis

**DOI:** 10.1371/journal.pmed.1000416

**Published:** 2011-02-15

**Authors:** Nicola Low, Matthew F. Chersich, Kurt Schmidlin, Matthias Egger, Suzanna C. Francis, Janneke H. H. M. van de Wijgert, Richard J. Hayes, Jared M. Baeten, Joelle Brown, Sinead Delany-Moretlwe, Rupert Kaul, Nuala McGrath, Charles Morrison, Landon Myer, Marleen Temmerman, Ariane van der Straten, Deborah Watson-Jones, Marcel Zwahlen, Adriane Martin Hilber

**Affiliations:** 1 Institute of Social and Preventive Medicine, University of Bern, Bern, Switzerland; 2 Centre for Health Policy, School of Public Health, University of Witwatersrand, Johannesburg, South Africa; 3 International Centre for Reproductive Health, Department of Obstetrics and Gynaecology, Ghent University, Ghent, Belgium; 4 Department of Infectious Disease Epidemiology and Clinical Research, London School of Hygiene and Tropical Medicine, London, United Kingdom; 5 Academic Medical Center of the University of Amsterdam and Amsterdam Institute for Global Health and Development, Amsterdam, The Netherlands; 6 Departments of Global Health, Medicine, and Epidemiology, University of Washington, Seattle, Washington, United States of America; 7 Department of Epidemiology, University of California, Los Angeles, California, United States of America; 8 Reproductive Health and HIV Research Unit, University of Witwatersrand, Hillbrow, South Africa; 9 Department of Medicine, University of Toronto, Toronto, Canada; 10 Africa Centre for Health and Population Studies, University of Kwa-Zulu Natal, Somkhele, South Africa; 11 Clinical Sciences Department, FHI, Research Triangle Park, North Carolina, United States of America; 12 Centre for Infectious Diseases Epidemiology and Research, School of Public Health & Family Medicine, University of Cape Town, Cape Town, South Africa; 13 Department of Epidemiology, Mailman School of Public Health, Columbia University, New York, New York, United States of America; 14 Women's Global Health Imperative, RTI International, San Francisco, California, United States of America; National Institute of Child Health and Human Development, United States of America

## Abstract

**Background:**

Identifying modifiable factors that increase women's vulnerability to HIV is a critical step in developing effective female-initiated prevention interventions. The primary objective of this study was to pool individual participant data from prospective longitudinal studies to investigate the association between intravaginal practices and acquisition of HIV infection among women in sub-Saharan Africa. Secondary objectives were to investigate associations between intravaginal practices and disrupted vaginal flora; and between disrupted vaginal flora and HIV acquisition.

**Methods and Findings:**

We conducted a meta-analysis of individual participant data from 13 prospective cohort studies involving 14,874 women, of whom 791 acquired HIV infection during 21,218 woman years of follow-up. Data were pooled using random-effects meta-analysis. The level of between-study heterogeneity was low in all analyses (*I* ^2^ values 0.0%–16.1%). Intravaginal use of cloth or paper (pooled adjusted hazard ratio [aHR] 1.47, 95% confidence interval [CI] 1.18–1.83), insertion of products to dry or tighten the vagina (aHR 1.31, 95% CI 1.00–1.71), and intravaginal cleaning with soap (aHR 1.24, 95% CI 1.01–1.53) remained associated with HIV acquisition after controlling for age, marital status, and number of sex partners in the past 3 months. Intravaginal cleaning with soap was also associated with the development of intermediate vaginal flora and bacterial vaginosis in women with normal vaginal flora at baseline (pooled adjusted odds ratio [OR] 1.24, 95% CI 1.04–1.47). Use of cloth or paper was not associated with the development of disrupted vaginal flora. Intermediate vaginal flora and bacterial vaginosis were each associated with HIV acquisition in multivariable models when measured at baseline (aHR 1.54 and 1.69, *p*<0.001) or at the visit before the estimated date of HIV infection (aHR 1.41 and 1.53, *p*<0.001), respectively.

**Conclusions:**

This study provides evidence to suggest that some intravaginal practices increase the risk of HIV acquisition but a direct causal pathway linking intravaginal cleaning with soap, disruption of vaginal flora, and HIV acquisition has not yet been demonstrated. More consistency in the definition and measurement of specific intravaginal practices is warranted so that the effects of specific intravaginal practices and products can be further elucidated.

*Please see later in the article for the Editors' Summary*

## Abstract 2

Pooling of data from 14,874 women in an individual participant data meta-analysis by Nicola Low and colleagues reveals that some intravaginal practices increase the risk of HIV acquisition.

## Editors' Summary

**Background:**

Since the first reported case of acquired immunodeficiency syndrome (AIDS) in 1981, the number of people infected with the human immunodeficiency virus (HIV), which causes AIDS, has risen steadily. By the end of 2009, an estimated 33.3 million people were living with HIV/AIDS. At the beginning of the epidemic, more men than women were infected with HIV but now, globally, more than half of all adults living with HIV/AIDS are women, and HIV/AIDS is the leading cause of death among women of child-bearing age. In sub-Saharan Africa, where more than two-thirds of HIV-positive people live, the situation for women is particularly bad. About 12 million women live with HIV/AIDS in this region compared with about 8 million men; among 15–24 year-olds, women are eight times more likely than men to be HIV-positive. This pattern of infection has developed because in sub-Saharan Africa most people contract HIV through heterosexual sex.

**Why Was This Study Done?:**

If modifiable factors that increase women's vulnerability to HIV infection could be identified, it might be possible to develop effective female-initiated prevention interventions. Some experts think that intravaginal practices such as cleaning the vagina with soap or a cloth increase the risk of HIV infection by damaging the vagina's lining or by increasing bacterial vaginosis (a condition in which harmful bacteria disrupt the healthy vaginal flora) but the evidence for such an association is inconclusive. In this meta-analysis, the researchers pool individual participant data from several prospective longitudinal cohort studies to assess the association between intravaginal practices and HIV acquisition among women in sub-Saharan Africa. Meta-analysis is a statistical method that combines data from several studies to get a clearer view of the factors associated with of a disease than is possible from individual studies. In a prospective longitudinal cohort study, groups of participants with different baseline characteristics (here, women who did or did not use intravaginal practices), who do not have the outcome of interest at the start of the study (here, HIV infection) are followed to see whether these characteristics affect disease development.

**What Did the Researchers Do and Find?:**

The researchers pooled individual participant data from 13 prospective cohort studies in sub-Saharan Africa involving nearly 15,000 women, 791 of whom acquired HIV, and asked whether HIV infection within 2 years of study enrollment was associated with self-reported intravaginal practices. That is, were women who used specific intravaginal practices more likely to become infected with HIV than women who did not use these practices? After controlling for age, marital status, and the number of recent sex partners, women who used cloth or paper to clean their vagina were nearly one and half times more likely to have acquired HIV infection as women who did not use this practice (a pooled adjusted hazard ratio [aHR] of 1.47). The insertion of products to dry or tighten the vagina and intravaginal cleaning with soap also increased women's chances of acquiring HIV (aHRs of 1.31 and 1.24, respectively). Moreover, intravaginal cleaning with soap was associated with the development of bacterial vaginosis, and disrupted vaginal flora and bacterial vaginosis were both associated with an increased risk of HIV acquisition.

**What Do These Findings Mean?:**

These findings suggest that some intravaginal practices increase the risk of HIV acquisition but they do not prove that there is a causal link between any intravaginal practice, disruption of vaginal flora, and HIV acquisition. It could be that the women who use intravaginal practices share other unknown characteristics that affect their vulnerability to HIV infection. The accuracy of these findings is also likely to be affected by the use of self-reported data and inconsistent definitions of intravaginal practices. Nevertheless, given the widespread use of intravaginal practices in some sub-Saharan countries (95% of female sex workers in Kenya use such practices, for example), these findings suggest that encouraging women to use less harmful intravaginal practices (for example, washing with water alone) should be included in female-initiated HIV prevention research strategies in sub-Saharan Africa and other regions where intravaginal practices are common.

**Additional Information:**

Please access these Web sites via the online version of this summary at http://dx.doi.org/10.1371/journal.pmed.1000416

- The US National Institute of Allergy and Infectious Diseases provides information on HIV infection and AIDS and on bacterial vaginosis
- The US Centers for Disease Control and Prevention has information on all aspects of HIV/AIDS, including specific information about HIV/AIDS and women; it also has information on bacterial vaginosis (in English and Spanish)
- HIV InSite has information on all aspects of HIV/AIDS
- Information is available from Avert, an international AIDS nonprofit on all aspects of HIV/AIDS, including HIV/AIDS and women and HIV/AIDS in Africa (in English and Spanish)
- A full description of the researchers' study protocol is available
- Several Web sites provide information on microbicides Global Campaign for Microbicides, Microbicides Development Programme, Microbicides Trials Network, and International Partnership for Microbicides

## Introduction

HIV-infected women outnumber men in many sub-Saharan African countries [1]. Novel female-initiated preventive methods have, so far, proved elusive [2],[3], so identifying new modifiable factors that affect women's vulnerability to HIV might help in the development of new preventive interventions [4]. Women use a wide range of products, applied in a variety of ways inside the vagina, to manage their sexual relationships, menstruation, and to improve wellbeing [5].

It has been hypothesised that some intravaginal practices could increase the risk of HIV infection by causing physical abrasions [4] or by disrupting the vaginal epithelium and increasing the occurrence of bacterial vaginosis (BV) [6]–[8]. An association between BV and HIV has been shown in cross-sectional and prospective studies [9],[10] and, more recently, intermediate grades of vaginal flora have also been associated with an increased risk of HIV acquisition [11]. However, the evidence linking intravaginal practices, BV, and HIV infection is currently inconclusive [8],[12],[13], as is evidence of associations between these practices and BV [12]. A recent systematic review found that few prospective studies recorded intravaginal practices consistently and that there was substantial heterogeneity between studies reporting associations between intravaginal practices and HIV [12]. Even large individual studies lack statistical power to examine the effects of specific intravaginal practices on HIV acquisition. Combining individual participant data from different studies might overcome some of these problems because data can be analysed consistently across studies and statistical power and precision can be increased [14]. Our overall aim was to determine whether specific vaginal practices increase the risk of a woman acquiring HIV infection by facilitating disturbances in vaginal flora or vaginal epithelial disruption. The primary objective of this study was to pool individual participant data from prospective longitudinal studies to investigate the association between intravaginal practices and acquisition of HIV infection among women in sub-Saharan Africa. Secondary objectives were to investigate associations between intravaginal practices and disrupted vaginal flora; and between disrupted vaginal flora and HIV acquisition.

## Methods

The study protocol specified hypotheses, inclusion criteria, and methods of analysis, and is available at http://www.ispm.ch/uploads/media/VP_IPD_protocol__final_090205_01.pdf. The review was reported according to the guidelines of the Preferred Reporting Items for Systematic Reviews and Meta-Analyses (PRISMA) group (Text S1).

### Study Selection

Potentially eligible studies were cohort studies and randomised controlled trials conducted in sub-Saharan Africa that had collected data prospectively about both intravaginal practices and incident HIV infection in females aged 10 y or older. We did not consider studies examining associations between disrupted vaginal flora and HIV unless data on intravaginal practices were also collected. We excluded studies in which participants were asked not to use intravaginal practices during the study or where the primary vaginal practice was female genital cutting or genital surgery. We also excluded studies involving vaginal microbicides or placebo products, tampons, or other devices to deliver medication. However, the control group in such studies was eligible for inclusion if they did not receive these interventions.

We identified studies using the results of a systematic review of published studies, which has been described elsewhere [12]. We obtained published reports of all studies included in the previous review and asked experts in the field to identify additional studies that had collected data about both intravaginal practices and incident HIV infections. We tried to contact the corresponding author or principal investigator of all potentially eligible studies by e-mail to determine whether relevant data had been collected and to invite them to contribute to the individual participant data meta-analysis. We included eligible studies for which we had received both a signed agreement to participate and a dataset by 16th March 2009. All included studies were approved by relevant country-specific and institutional ethical review boards and all participants within each study provided written informed consent for the original studies.

### Data Collection

We used protocols, questionnaires, and publications from included studies, and information from investigators to determine whether requested variables had been collected or could be derived. The final variable list included: HIV infection, intravaginal practices, vaginal flora status, herpes simplex virus infection (HSV-2) status, age, education, religion, marital status, employment, age at first sex, numbers of sex partners, sex in exchange for money, pregnancy and contraception, and general condom use (consistent, inconsistent, or never). A named investigator for each included study provided access to the dataset, answered questions about the study procedures and coding of variables, and contributed to interpretation of results and revising the manuscript. For most studies we obtained raw data and recoded these in house (MFC, KS, or SCF). For two studies, a data manager provided a dataset, coded according to our predefined scheme.

### Outcome and Exposure Measures

The primary outcome was HIV infection diagnosed within the first 2 y, using the diagnostic criteria defined by each individual study. We included women with a negative HIV test at the baseline visit and at least one follow-up HIV test and estimated the date of HIV infection as the midpoint between the last negative and the first positive test.

Intravaginal practices were the main exposures and were based on self-reported data collected in face-to-face interviews in all but one study, which instead used audio computer-assisted self-interviewing [15]. Definitions were based on a published classification system [6]. After examining the data available in individual studies, we defined five separate intravaginal practices (Box 1). We used baseline data about current practices (that is, those from the 1 to 3 mo preceding the start of the study), since there were too few studies reporting repeated measures of these variables to consider changes in practices over time. The reference group was women not at increased risk of acquiring HIV infection through intravaginal practices, and included women using no intravaginal practice or water.

**Box 1. Definitions of intravaginal practices used in this study**

**Intravaginal Practice Definition**

**Cleaning with water** Cleaning inside the vagina, beyond the introitus, with water as the only product. Can be with or without specific mention of fingers, other materials, or douching devices to introduce water inside the vagina.

**Cleaning with soap** Cleaning inside the vagina, beyond the introitus, with generic “soap” or “household soap,” or named proprietary bath soaps. Can be with or without specific mention of fingers, other materials, or douching devices to introduce soap lather inside the vagina.

**Cleaning with other household products** 

Cleaning inside the vagina, beyond the introitus, with products that include: generic “household cleaners”; named proprietary products such as “Omo”; antiseptic solutions; vinegar; lemon juice. Can be with or without specific mention of fingers, other materials, or douching devices to introduce liquid inside the vagina.

**Cloth to wipe out vagina or apply products** 

Use inside the vagina, beyond the introitus, of materials such as cloth, tissue, paper, cotton wool to wipe out vaginal secretions or to apply products. Includes specific practices described as “cleaning with cloth” without any other product and named products introduced with cloth or other material. Does not include use of medication, tampons, or removal of menstrual blood.

**Insertion of products to dry or tighten vagina** 

Pushing or placing mostly nonliquid products inside the vagina (including powders, creams, herbs, tablets, sticks, stones, leaves, “traditional products”) regardless of the duration. Some questions ask specifically about the use of this practice before sexual intercourse. The intention is to achieve a sensation described as dry or tight.

**Any (or no) current practice** 

Includes all positive (or negative) responses to general questions about the use of an intravaginal practice, or to specific questions about practices described above. Time period is that asked about at the baseline visit, usually past 1–3 mo.

Categories are not mutually exclusive. Definitions of intravaginal cleaning and insertion adapted from classification developed by the WHO Gender, Sexuality and Vaginal Practices Study Group (GSVP Study Group) [6]. Additional definitions based on specific questions used in individual studies.

Vaginal flora status was considered as both an exposure and an outcome, and assessed using scores from Gram-stained vaginal smears [16],[17], or Amsel clinical criteria [18]. We used the Nugent score if results from more than one method were available [16]. BV was defined as a Nugent score of 7–10, Ison-Hay grade III, or the presence of three or more Amsel criteria. Intermediate vaginal flora was defined as a Nugent score of 4–6 [11] or Ison-Hay grade II. Two studies used Amsel criteria only and could not be included in analyses that included intermediate vaginal flora as an exposure or outcome [19],[20].

### Assessment of the Risk of Bias

We assessed the potential for bias in each cohort study, arising from prespecified methodological domains [21]: description of participation and evidence of bias; definitions of diagnostic criteria; blinding of outcome assessment; conduct of follow-up; missing data; and measurement of main confounders.

### Statistical Analysis

#### Association between intravaginal practices and HIV infection

For the primary objective we included all women and used Cox proportional hazards models to examine associations in each study between each intravaginal practice at baseline and HIV acquisition, and expressed these as the hazard ratio (HR) and 95% confidence intervals (CIs). Follow-up time was measured until the first of: estimated date of HIV infection; the last follow-up visit; the end of the study; or after 2 y of follow-up. We pooled data if the level of between-study heterogeneity was mild or moderate, defined as a value from the *I* ^2^ statistic below 50% [22]. We used two methods [14]: for all study objectives we used random effects meta-analysis to combine effect estimates from individual strata (two-stage method); for analyses with HIV acquisition as the outcome we also used stratified, fixed effects Cox regression, with studies as the strata (one-stage method). Both methods gave very similar results. The proportional hazards assumption, tested on the basis of Schönfeld residuals, was not violated in any model. All statistical analyses were conducted using Stata (version 10, Stata Corporation).

We looked for statistical evidence of confounding of the association between intravaginal practices and HIV infection in each individual study and for each intravaginal practice by comparing the univariable HR with the HR from bivariable models including baseline measures of each of the following prespecified factors: marital status; numbers of sex partners; condom use; contraception; educational level; religion; employment; age at first intercourse; having received goods or money in exchange for sex; pregnancy; and HSV-2 status. If inclusion of the variable resulted in the HR changing by more than 10% in one or more individual studies we considered it for inclusion in multivariable analyses [23]. We controlled for age in all multivariable models. We present results from models that also controlled for marital status and number of sex partners during the previous 3 mo as recorded at cohort entry because these were the variables that fulfilled our criteria most often. The results were very similar to those from models that controlled for a larger common set of variables, or controlled for different variables in each study.

We then examined the role of BV. We aimed to control for time-varying confounding by BV [24], but there were too many differences between studies in the frequency and timing of vaginal specimen collection to use fully time-updated measures. We therefore conducted two exploratory analyses in multivariable models to see, qualitatively, whether there was attenuation of the HR; using either the result of the baseline test or the last available test for BV before the estimated date of HIV infection, or before the date of censoring.

#### Associations between intravaginal practices and changes in vaginal flora status

To examine associations between intravaginal practices and short-term changes in vaginal flora status we included women who had normal vaginal flora at baseline, assessed using Gram-stain criteria, and a follow-up assessment within the first year of enrolment. We categorised vaginal flora status at follow-up as normal, intermediate, or BV. We used ordered logistic regression with a proportional odds model [25]. The assumption of the model is that the odds ratio (OR) associated with an intravaginal practice for the odds of intermediate vaginal flora or BV compared with normal vaginal flora is equal to the OR for BV compared with normal or intermediate flora. The model therefore estimates a single OR from the data.

#### Association between disrupted vaginal flora and HIV acquisition

To examine the association between disrupted vaginal flora and HIV acquisition we included all women with vaginal flora assessed by Gram-stain at baseline. We considered vaginal flora status as a three-level ordered exposure and used Cox proportional hazards regression to estimate the HR (and 95% CI) for incident HIV infection in the first 2 y of follow-up.

## Results

### Description of Studies

**Figure 1:**
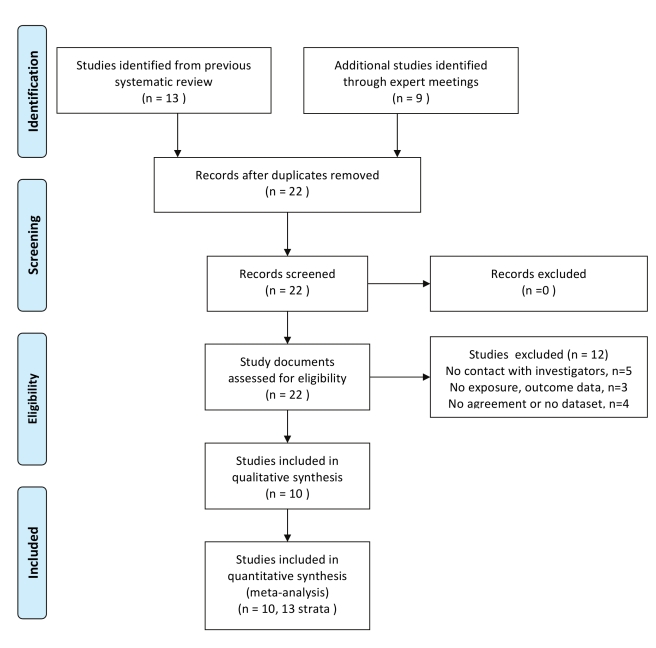
Flow diagram of included studies.

We assessed 22 studies for eligibility; 13 prospective studies conducted in sub-Saharan Africa included in our previous systematic review [8],[19],[20],[26]–[35] and nine identified through expert meetings [15],[36]–[43]. We excluded five studies for which we could not determine eligibility because we did not establish contact with the authors [26]–[28],[31],[33], three studies that did not include data on relevant exposures or outcomes [30],[32],[34], and four studies for which the investigators declined to take part, or could not send their agreement to participate or dataset by 16th March 2009 (Figure 1; Table S1) [29],[38]–[40]. We included ten studies [8],[15],[19],[20],[35]–[37],[41]–[43]. We analysed the data as 13 separate studies, stratifying results from three multicentre studies according to enrolment site [15],[19],[35]. Table 1 shows selected characteristics of the studies, which were done in six sub-Saharan African countries and included data from 14,874 women followed for 21,218 woman years, with 791 incident HIV infections in the first 2 y. The individual studies were generally assessed as having a low risk of bias (data available on request). Most studies involved women enrolled from community settings or clinics providing reproductive health services (studies 1, 6, 7, 8, 9, 10). Two studies in Tanzania enrolled women working as food sellers or in bars and other recreational facilities, amongst whom high prevalences of sexually transmitted infections and HIV have previously been reported (studies 4, 5). Two studies in Kenya enrolled only women who were self-identified sex workers (studies 2, 3). The mean age at enrolment was 29.4 y (standard deviation [SD] 8.7, range 15.2–67.0 y). The incidence of HIV infection in individual studies ranged from 1.6 (95% CI 1.2–2.0, study 1 Uganda) to 15.1 (95% CI 10.5–21.8, study 9) per 100 woman years. The median time from enrolment to the estimated date of HIV infection was 250 d (interquartile range [IQR] 130–415 d).

**Table 1:** Characteristics of participants of individual cohort studies.

Study Number; Country [Reference]	Enrolment Settings	Study Population	Planned Study Duration^a^	Planned Frequency of Follow-up^a^	Dates of Enrolment^a^	*n* Included^b^	Age at Enrolment (y), Mean (SD)	Follow-up per Woman (mo), Median (IQR)	Percent Followed for 12 mo^c^	*n* Incident HIV Infections	HIV Incidence, per 100 Woman Years (95% CI)
1; Uganda [35]	FPC and other health services	Women attending clinics	15–24 mo	3 Monthly	11/99–03/02	2,201	24.9 (4.5)	23.7 (21.4–24.0)	97.0	63	1.6 (1.2–2.0)
2; Kenya [37]	Randomised trial (all women); peer-leaders network	Self-identified female sex workers	24 mo	6 Monthly	05/98–01/02	414	29.1 (8.0)	23.8 (11.2–24.0)	75.4	30	4.9 (3.4–7.0)
3; Kenya [8]	Municipal STI clinic	Self-identified female sex workers attending a STI clinic	Not fixed	Monthly	02/93–12/02	1,270	27.1 (6.3)	14.3 (3.7–24.0)	55.1	164	11.3 (9.7–13.2)
4; Tanzania [42]	Reproductive health clinics in selected guesthouses	Women working in bars, restaurants, guesthouses	12 mo	3 Monthly	08/02–10/03	978	30.0 (8.2)	11.7 (8.7–12.0)	66.0	23	2.8 (1.9–4.3)
5; Tanzania [43]	Randomised trial (all women); mobile health clinics	Women working in bars, restaurants, guesthouses	30 mo	3 Monthly	11/03–01/06	781	27.5 (5.0)	24.0 (15.5–24.0)	91.3	45	3.4 (2.6–4.6)
6; Malawi [19]	FPC, postnatal clinics	Women attending clinics	9 mo	3 Monthly	06/01–08/02	993	27.9 (8.2)	9.0 (8.7–9.1)	67.5^c^	33	4.9 (3.5–6.9)
6; Zimbabwe [19]	As above	As above	As above	As above	As above	526	29.0 (8.4)	9.0 (8.8–9.2)	74.7^c^	19	5.2 (3.3–8.1)
1; Zimbabwe [35]	FPC and other health services	Women attending clinics	15–24 mo	3 Monthly	11/99–08/02	2,248	25.9 (4.4)	23.0 (17.3–24.0)	90.7	153	4.2 (3.6–4.9)
7; Zimbabwe, [15]	Randomised trial (control arm only); FPC, well-baby, general health clinics; community organisations	Sexually active women (average four sex acts per month)	12–24 mo	3 Monthly	09/03–10/05	1,229	28.4 (7.2)	23.5 (17.9–23.9)	96.2	52	2.5 (1.9–3.3)
7; South Africa [15]	As above	As above	As above	As above	As above	1,247	28.9 (8.0)	17.9 (14.4–22.3)	91.9	151	5.5 (4.5–6.7)
8; South Africa [36]	FPC, well-baby, postnatal clinics	Women attending clinics	12 mo	6 Monthly	01/02–01/04	694	24.7 (5.0)	11.0 (10.8–11.4)	74.1	20	3.4 (2.2–5.3)
9; South Africa [41]	FPC, immunisation clinics	Women attending clinics	12 mo	3 Monthly	07/03–07/04	261	29.3 (9.5)	9.5 (6.0–12.0)	42.9	29	15.1 (10.5–21.8)
10; South Africa [20]	Cervical cancer trial; community meetings and health workers	Women never screened for cervical cancer living in Khayelitsha	6–36 mo	3 Monthly	08/01–11/03	2,032	43.2 (6.8)	24.0 (24.0–24.0)	90.9	61	1.7 (1.3–2.2)

Number of incident HIV infections during up to 2 y follow-up (n = 14,874), ordered geographically from north to south.

a From study protocol or publication.

b Includes only women who were HIV negative at start of follow-up and had at least one follow-up visit.

c Includes women who attended a follow-up visit at 12 mo ±30 d, except study 6, which includes women who completed study follow-up at 9 mo.

FPC, family planning clinic; SD, standard deviation; STI, sexually transmitted infections.

### Frequency of Intravaginal Practices

The percentage of women reporting any current intravaginal practice at baseline ranged from 18% (study 8) to 95% (study 3, Table 2). Studies done in South Africa tended to have a low overall prevalence of any current vaginal practice (18%–27%, studies 8–10) and studies in Zimbabwe tended to have a high prevalence (69%–92%, studies 1, 6, 7). Female sex workers in Kenya (76%–95%, studies 2, 3) and high risk women in Tanzania (67%–76%, studies 5, 4) also reported high levels of any vaginal practice. Table 2 shows the proportions of women reporting any use of specific practices, i.e., whether or not they reported other practices. Cleaning inside the vagina with soap was the most common practice involving a specified product, and was reported by more than one-third of women in six studies in the most northern countries in the region (studies 1–6 Malawi). Reported use of other more abrasive household cleaning products was uncommon, ranging from 0.7% (study 8) to 7% (study 2). Reported use of cloth or paper to wipe out the vagina, or apply products, ranged from 0.3% (study 8) to 70% (study 7 Zimbabwe). Inserting products to dry or tighten the vagina was uncommon; this was most commonly reported in four studies conducted in South Africa and Zimbabwe, where the prevalence was 13%–20% (studies 1 Zimbabwe, 7 Zimbabwe, and 8 South Africa). Cleaning with water, with or without other practices, was reported by more than 60% of women in all but four studies in South Africa (studies 7 South Africa, 8–10). Where measurements of intravaginal practices were available at follow-up visits, the majority of reported practices were consistent with baseline data; 60% reported the same practice at all study visits at which data were collected, 34% reported either the same practice or no practice, and 6% reported different practices at all visits.

**Table 2:** Baseline prevalence of intravaginal practices and BV in included cohort studies (*n = *14,874).

Study Number; Country	*n* Included	Any Practice^a^	Specific Practices						*n* BV (%)
		*n* Yes (%)	*n* Cleaning with Water Only (%)^b^	*n* Cleaning with Water (%)^c^	*n* Cleaning with Soap (%)^c^	*n* Cleaning with Household Products (%)^c^	*n* Use of Cloth, Tissue, Paper (%)^c^	*n* Insertion to Dry/Tighten (%)^c^	
1; Uganda	2,201	1,510 (68.6)	500 (22.7)	1,492 (67.8)	982 (44.6)	32 (1.5)	195 (8.9)	30 (1.4)	465 (21.1)
2; Kenya	414	323 (78.0)	Not asked	273 (65.9)	207 (50.0)	30 (7.3)	2 (0.5)	Not asked	197 (47.6)
3; Kenya	1,270	1,204 (94.8)	243 (19.1)	1,066 (83.9)	820 (64.6)	85 (6.7)	187 (14.7)	11 (0.9)	461 (36.3)
4; Tanzania	978	740 (75.7)	372 (38.0)	728 (74.4)	348 (35.6)	8 (0.8)	32 (3.3)	26 (2.7)	446 (45.6)
5; Tanzania	781	523 (67.0)	101 (11.1)	499 (63.9)	397 (50.8)	16 (2.1)	32 (4.1)	33 (4.2)	482 (61.7)
6; Malawi	993	891 (89.7)	182 (18.3)	880 (88.6)	669 (67.4)	23 (2.3)	47 (4.7)	33 (3.3)	77 (7.8)
6; Zimbabwe	526	364 (69.2)	262 (49.8)	356 (67.7)	68 (12.9)	18 (3.4)	29 (5.5)	6 (1.1)	85 (16.2)
1; Zimbabwe	2,248	1,916 (85.2)	1,323 (58.9)	1,724 (76.7)	320 (14.2)	106 (4.7)	1,122 (49.9)	445 (19.8)	639 (28.4)
7; Zimbabwe	1,229	1,127 (91.7)	629 (51.2)	810 (65.9)	166 (13.5)	41 (3.3)	854 (69.5)	154 (12.5)	82 (6.7)^d^
7; South Africa	1,247	1,123 (90.1)	86 (6.9)	668 (53.6)	582 (46.7)	49 (3.9)	655 (52.5)	164 (13.2)	0^d^
8; South Africa	694	124 (17.9)	19 (2.7)	44 (6.3)	17 (2.5)	5 (0.7)	2 (0.3)	87 (12.5)	349 (50.3)
9; South Africa	261	68 (26.1)	19 (7.3)	56 (21.5)	36 (13.8)	14 (5.4)	37 (14.2)	8 (3.1)	124 (47.5)
10; South Africa	2,032	546 (26.9)	304 (15.0)	460 (22.6)	103 (5.1)	85 (4.2)	438 (21.6)	154 (7.6)	174 (8.6)

a Includes any intravaginal practice reported at the baseline visit.

b Water is the only substance put into the vagina and woman does not use any other intravaginal practice (this category was grouped with “no intravaginal practice” to form the reference category for comparative analyses).

c Woman reports using this practice and may or may not report any other intravaginal practice.

d Based on a subset of 257 women tested for BV at baseline in the Zimbabwe study site only.

### Associations between Intravaginal Practices and HIV Infection

**Figure 2 pmed-1000416-g002:**
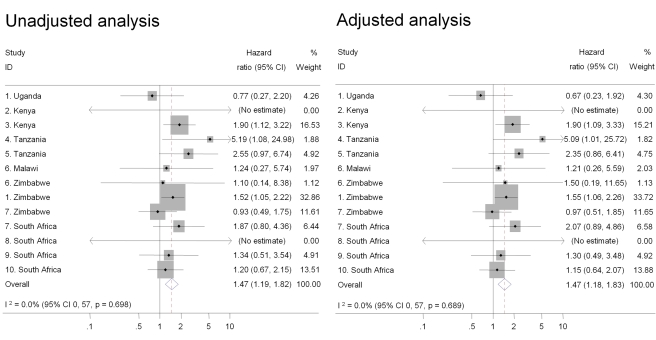
Association between use of cloth or paper to wipe out vagina or apply products and HIV acquisition, ordered by country, north to south (*n* = 10,332). Individual study results from Cox regression. Pooled unadjusted and aHRs from random effects meta-analysis. Reference group is women who reported no intravaginal practice or cleaning with water only. Multivariable models adjusted for age, marital status, and number of partners in last 3 mo. No estimate possible if there were no events in one group.

**Figure 3 pmed-1000416-g003:**
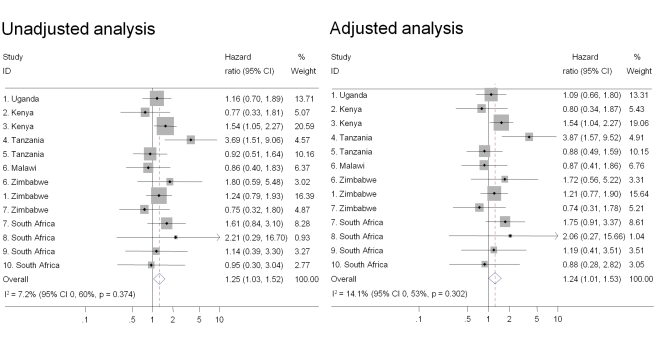
Association between intravaginal cleaning with soap and HIV acquisition, ordered by country, north to south (*n* = 3,071). Individual study results from Cox regression. Pooled unadjusted and aHRs from random effects meta-analysis. Reference group is women who reported no intravaginal practice or cleaning with water only. Multivariable models adjusted for age, marital status, and number of partners in last 3 mo.

**Figure 4:**
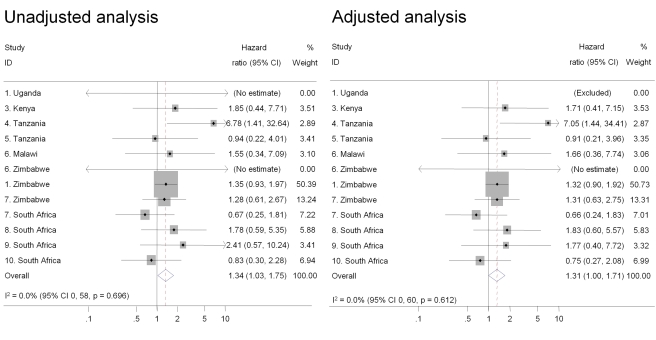
Association between insertion of products to dry or tighten the vagina and HIV acquisition, ordered by country, north to south(*n* = 9,420). Individual study results from Cox regression. Pooled unadjusted and aHRs from random effects meta-analysis. Reference group is women who reported no intravaginal practice or cleaning with water only. Multivariable models adjusted for age, marital status, and number of partners in last 3 mo. No estimate possible if there were no events in one group. Stratum excluded if there were no events in either group, or standard error could not be estimated by model.

**Figure 5:**
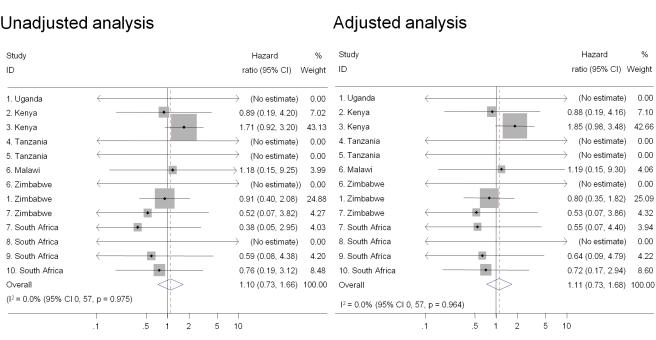
Association between intravaginal cleaning with household cleaners and HIV acquisition, ordered by country, north to south (*n* = 8,879). Individual study results from Cox regression. Pooled unadjusted and aHRs from random effects meta-analysis. Reference group is women who reported no intravaginal practice or cleaning with water only. Multivariable models adjusted for age, marital status, and number of partners in last 3 mo. No estimate possible if there were no events in one group.

Intravaginal use of cloth or paper to wipe out the vagina or apply products was the practice most strongly associated with HIV acquisition in univariable analysis and after controlling for age, marital status, and number of sex partners in the past 3 mo (pooled adjusted HR 1.47, 95% CI 1.18–1.83) (Figure 2). There was also an increased risk of HIV acquisition in women reporting intravaginal cleaning with soap and water (pooled adjusted HR 1.24, 95% CI 1.01–1.53) (Figure 3) and insertion of products to dry or tighten the vagina (pooled adjusted HR 1.31, 95% CI 1.00–1.71) (Figure 4). One study (study 2) did not ask about insertion practices. The use of household cleaning products other than soap was much less common; in four studies the HR could not be estimated (studies 1 Uganda, 4, 5, 8; Figure 5). In the remaining studies the pooled analyses showed no evidence of an increased risk of HIV acquisition (pooled adjusted HR 1.11, 95% CI 0.73–1.68). There was little evidence of between-study heterogeneity in any of these analyses (*I* ^2^ values 0%–14%). Results from the fixed effects models were the same or very similar to those from random effects models (Table S2).

There was no statistical evidence that intravaginal use of water alone increased the risk of HIV acquisition when compared with no practice in univariable (Table S3, pooled unadjusted HR 1.02, 95% CI 0.78–1.35, *I* ^2^ 3.9%) or multivariable analyses (pooled adjusted HR 1.03, 95% CI 0.76–1.40, *I* ^2^ 16.1%). Women who used only water intravaginally, compared with those using no practice, were younger (*p<*0.001), more likely to be currently married (*p<*0.001), more likely to have one partner in the last 3 mo (*p<*0.001), more likely to be using contraception (*p<*0.001), more likely to be Catholic or Muslim than Protestant (*p<*0.001), and less likely to never use condoms (*p = *0.005). Controlling for these variables did not alter the strength of the associations found in univariable analyses (unpublished results).

Table 3 shows the results of analyses controlling for BV in addition to demographic and behavioural characteristics. These analyses exclude most participants from study 7, in whom vaginal flora was only assessed in a subset of women from one study site. Intravaginal use of cloth or paper remained associated with HIV acquisition after controlling for BV. For cleaning with soap and insertion of products to dry or tighten the vagina, the associations with HIV acquisition were weakened slightly after adjustment for demographic and behavioural variables. Adjustment for the presence of BV at baseline or at the last visit before the estimated date of HIV infection did not have any additional effect. When vaginal flora status was considered with intermediate vaginal flora and BV as separate categories, the pattern of results was similar to that seen when BV was included as a binary variable, but confidence intervals were wider because studies that used only Amsel criteria for diagnosis were excluded (unpublished results).

**Table 3:** Associations between intravaginal practices and HIV acquisition, adjusting for different measures of disrupted vaginal flora.

Intravaginal Practice	Number in Model (Strata/Studies)^a^	HR (95% CI)		aHR (95% CI)					
		Unadjusted	*I* ^2^% (95% CI)	Demographic/Behavioural Factors^b^	*I* ^2^% (95% CI)	Demographic/Behavioural Factors + BV^c^ at Baseline	*I* ^2^% (95% CI)	Demographic/Behavioural Factors + BV^‡^ before Seroconversion	*I* ^2^% (95% CI)
Cleaning with soap and water	11,387 (12/10)	1.20 (0.97–1.49)	8.2 (0–62)	1.18 (0.94–1.48)	14.3 (0–54)	1.18 (0.94–1.49)	15.8 (0–55)	1.18 (0.94–1.48)	13.6 (0–53)
Cleaning with household products	7,893 (12/10)	1.20 (0.78–1.85)	0.0 (0–58)	1.19 (0.77–1.84)	0.0 (0–60)	1.25 (0.80–1.94)	0.0 (0–60)	1.18 (0.76–1.83)	0.0 (0–62)
Cloth to wipe out vagina or apply products	8,475 (12/10)	1.44 (1.13–1.82)	0.0 (0–58)	1.38 (1.06–1.80)	6.5 (0–61)	1.39 (1.06–1.81)	6.9 (0–61)	1.38 (1.03–1.85)	15.9 (0–56)
Insertion of products to dry or tighten vagina	8,216 (11/9)	1.36 (1.01–1.85)	0.0 (0–60)	1.32 (0.97–1.79)	0.0 (0–60)	1.33 (0.98–1.82)	0.0 (0–60)	1.32 (0.97–1.80)	0.0 (0–62)

HRs from two-stage random effects meta-analysis. Intravaginal practices measured at baseline; reference category is no vaginal practice or use of water only.

a Numbers of observations differ from Figures 1–4 because they exclude those with no BV measurement, mostly from study 7 in which BV was measured only in a subset at baseline in the Zimbabwe site; model for insertion of products also excludes study 2, which did not ask about this practice.

b Adjusted for age, marital status, and reported number of sex partners in last 3 mo, as recorded at cohort entry.

c BV as binary variable defined as Nugent score of 7–10, Ison-Hay grade III, or the presence of three or more Amsel criteria.

### Associations between Intravaginal Practices and Disrupted Vaginal Flora

Table 4 shows the associations between intravaginal practices and the development of intermediate vaginal flora or BV amongst women with normal vaginal flora at the baseline visit and vaginal specimens examined by Gram-stain criteria at follow-up. Amongst women who cleaned intravaginally with soap and water, the incidence of disrupted vaginal flora at the next visit was increased in univariable and multivariable analyses (pooled adjusted OR from ordered logistic regression 1.24, 95% CI 1.04–1.47). There was a similar but weaker trend for the insertion of products to dry or tighten the vagina, but confidence intervals for these estimates were wider (pooled adjusted OR 1.29, 95% CI 0.98–1.71, *p = *0.072). There was no evidence of an association between intravaginal cleaning with household products or use of cloth or paper and development of disrupted vaginal flora. There was no evidence of between-study heterogeneity (*I* ^2^ values 0.0% for all analyses).

**Table 4:** Associations between intravaginal practices and disrupted vaginal flora in women with normal vaginal flora at baseline.

Intravaginal Practice^a^	Number in Model (Strata/Studies)^b^	Number Developing Disrupted Flora^c^	Disrupted Vaginal Flora^b^			
			Unadjusted OR (95% CI)	*p*-Value	Adjusted OR (95% CI)^d^	*p*-Value
Cleaning with soap and water	3,222 (8/7)	1,088	1.27 (1.07–1.50)	0.006	1.24 (1.04–1.47)	0.014
Cleaning with household products	2,045 (7/6)	641	0.95 (0.62–1.44)	0.797	0.89 (0.58–1.36)	0.576
Cloth to wipe out vagina or apply products	2,177 (5/4)	704	1.06 (0.85–1.32)	0.588	1.06 (0.85–1.33)	0.577
Insertion of products to dry or tighten vagina	2,264 (7/6)	735	1.26 (0.96–1.66)	0.099	1.29 (0.98–1.71)	0.072

OR from two-stage random effects meta-analysis based on ordered logistic regression.

a Baseline category for intravaginal practices is no vaginal practice or use of water only. Intravaginal practices measured at baseline.

b Disrupted vaginal flora as a three-level ordered categorical variable: normal vaginal flora defined as Nugent score 0–3, or Ison-Hay grade I; intermediate vaginal flora defined as Nugent score 4–6, or Ison-Hay grade II; BV defined as Nugent score 7–10, or Ison-Hay grade III. Excludes two studies that did not use Gram stain criteria [19],[20].

c Number with normal flora at baseline who developed disrupted vaginal flora includes both women using and not using each intravaginal practice.

d Adjusted for age, marital status, and reported number of sex partners in last 3 mo as reported at cohort entry.

### Associations between Disrupted Vaginal Flora and HIV Infection

Vaginal flora status was associated with HIV incidence in univariable and multivariable analyses (Table 5). The risk of HIV acquisition was higher in women with BV than with intermediate vaginal flora. Controlling for potential confounders did not substantially attenuate the effect estimates. The strength of association between vaginal flora status and HIV acquisition was slightly weaker when using the vaginal flora status measured at the visit preceding the estimated date of HIV infection (median 51 d, IQR 40–147 d between vaginal flora assessment and HIV infection) than when using the baseline value (median 253 d, IQR 116–426 d).

**Table 5:** Association between disrupted vaginal flora and HIV acquisition, stratified Cox regression.

Variable	Baseline Vaginal Flora Status (*n* = 8,452)^a^			Vaginal Flora Status at Visit before HIV Seroconversion (*n* = 8,626)^a^		
	Unadjusted HR (95% CI)	Adjusted HR (95% CI)^b^	*p*-Value	Unadjusted HR (95% CI)*	Adjusted HR (95% CI)^b^	*p*-Value
Vaginal flora			<0.001			<0.001
Normal vaginal flora	1 (reference)	1 (reference)		1 (reference)	1 (reference)	
Intermediate vaginal flora	1.62 (1.27–2.08)	1.54 (1.20–1.97)		1.51 (1.19–1.91)	1.41 (1.12–1.79)	
BV	1.84 (1.48–2.28)	1.69 (1.36–2.10)		1.66 (1.35–2.05)	1.53 (1.24–1.89)	
HSV status at baseline						
Negative	1 (reference)			1 (reference)		
Positive	2.14 (1.70–2.70)	2.29 (1.80–2.90)	<0.001	2.14 (1.70–2.69)	2.31 (1.82–2.91)	<0.001
Age at cohort entry			<0.001			<0.001
>25 y	1.25 (1.04–1.50)	1.37 (1.13–1.65)		1.26 (1.05–1.52)	1.38 (1.14–1.66)	
25–34 y	1 (reference)	1 (reference)		1 (reference)	1 (reference)	
35 y or older	0.80 (0.56–1.15)	0.80 (0.56–1.15)		0.79 (0.55–1.13)	0.78 (0.54–1.12)	
Marital status			<0.001			<0.001
Currently married	1 (reference)	1 (reference)		1 (reference)	1 (reference)	
Currently unmarried	1.96 (1.46–2.64)	1.78 (1.32–2.40)		1.96 (1.46–2.62)	1.77 (1.31–2.38)	
Number of partners last 3 mo			0.034			0.023
No partner	0.97 (0.48–1.97)	0.94 (0.46–1.91)		0.96 (0.47–1.95)	0.90 (0.44–1.84)	
1 partner	1 (reference)	1 (reference)		1 (reference)	1 (reference)	
More than 1 partner	2.14 (1.47–3.12)	1.59 (1.09–2.31)		2.15 (1.48–3.13)	1.62 (1.11–2.35)	

a Included in analysis are women with available vaginal flora status measured by Gram stain criteria: normal vaginal flora defined as Nugent score 0–3, or Ison-Hay grade I; intermediate vaginal flora defined as Nugent score 4–6, or Ison-Hay grade II; BV defined as Nugent score 7–10, or Ison-Hay grade III. Excludes two studies that did not use Gram stain criteria [19],[20].

b Multivariable model controls for all variables in the table.

## Discussion

This study combined individual participant data from ten prospective studies in six sub-Saharan African countries. Intravaginal use of cloth or paper remained associated with HIV acquisition after controlling for age, marital status, number of sex partners in the past 3 mo, and in models that controlled for BV. Insertion of products to dry or tighten the vagina and intravaginal cleaning with soap were associated with HIV acquisition in univariable and multivariable analyses controlling for demographic and behavioural variables, but not in models that controlled for BV. Intravaginal cleaning with soap was also associated with the development of intermediate vaginal flora and BV at follow-up in women with normal vaginal flora at baseline. Disrupted vaginal flora measured at baseline or at the visit before the estimated data of HIV infection was associated with HIV acquisition in both univariable and multivariable analyses.

### Strengths and Weaknesses

The main strength of this study was the collaboration of investigators from ten different studies, which allowed the collation of individual participant data from nearly 15,000 women and analysis using consistent definitions of intravaginal practices across studies. By pooling data we increased the power and precision of our analyses and adjusted for confounding, which is difficult or impossible in an aggregate data meta-analysis [14]. There was a striking lack of between-study heterogeneity in results, despite differences in study populations, designs, and questionnaires, which increases the robustness of our findings. In addition, we obtained very similar results using different statistical methods to pool data. Our results might be biased because we did not identify or include all eligible studies. We did, however, conduct a wide-ranging search and reasons for exclusion were not related to study results.

Limitations of this study were mainly due to data collection differences that could not be remedied by recoding. Questions about intravaginal practices were asked in different ways because there are no agreed upon definitions [5], there is no validated measurement instrument, and the purposes of the studies differed. We therefore had to limit the number of specific intravaginal practices from those originally planned and differences in wording of questions about intravaginal practices between studies might have affected our results. By grouping exposure categories, we might have masked harms (or benefits) of particular practices or products. Grouping of categories to obtain uniformly defined variables for confounding factors, or imprecision in the measurement of other variables included in the analysis might also have resulted in residual confounding. In addition, we cannot exclude the possibility of residual confounding from unmeasured factors, such as sexually transmitted infections, from our reliance on baseline measures of intravaginal practices that changed over time, or the motivation for performing certain practices, which can vary according to perceptions of risk [5].

A further limitation was the difficulty in definitively establishing the temporal sequence of intravaginal practices and changes in vaginal flora status; intravaginal practices could promote disruption of vaginal flora but symptoms related to those changes, such as vaginal discharge or fishy odour, could prompt intravaginal washing or wiping [24]. We tried to overcome this problem when examining the association between intravaginal practices and disrupted vaginal flora by including only women with normal vaginal flora at baseline. We could not, however, consider changes in exposure status over time because of data collection differences and uncertainty about the effects of treatment for BV, which was documented in some studies but not in others.

### Comparison with Other Studies

This study is likely to be the largest to have examined associations between intravaginal practices and HIV acquisition and it allowed us to reexamine some previously observed inconsistencies between published studies. For example, McClelland and colleagues found a strong association between intravaginal cleaning with soap and incident HIV infection in Kenya [8] but van de Wijgert and colleagues and Myer and colleagues found no associations in their studies in Uganda and Zimbabwe [44] and South Africa [20]. In this analysis, estimates from the same studies were close to the published data; when pooled with the other studies, using the same definitions and multivariable model, the results were consistent with the overall finding of a modest increase in the risk of HIV acquisition (Figures 1–4). Differences in definitions of intravaginal practices and methods of analysis in individual studies make it difficult to compare findings directly and to synthesise results across studies. These differences meant that in our systematic review of aggregated published data we were limited to examining associations with intravaginal cleaning and insertion of products and could not draw conclusions about any specific practice [12], illustrating the advantages of individual participant data meta-analysis.

Our findings about the associations between disrupted vaginal flora and increased risk of HIV are consistent with those of other studies [10],[11]. Given the rapid fluctuations that occur in vaginal microflora [11],[45], the intervals between assessment of vaginal flora status and HIV acquisition in our analyses, and those of previous studies, likely resulted in substantial misclassification. However, these analyses captured some of the increase in risk of HIV infection for women who had BV identified on at least one occasion. A disadvantage of our analysis is that this was a secondary objective and our search strategy did not include all studies addressing these associations. Nevertheless, we included more prospective studies than the previously published systematic review examining the links between BV and risk of HIV infection [10] and we were able to conduct both univariable and multivariable analyses across all studies.

### Interpretation of Study Findings

Our findings suggest an increase in the risk of acquiring HIV infection amongst women who use cloth or paper to wipe out the vagina or apply products, insert products intended to dry or tighten the vagina, or clean with soap intravaginally. Whilst effects of this magnitude could result from residual confounding or bias, there are also plausible biological mechanisms for these associations. Use of cloth or paper to wipe out the vagina was associated with HIV acquisition after controlling for BV and was not associated with the development of disrupted flora. Use of cloth might increase the risk of HIV if removal of protective vaginal mucus exposes existing micro-trauma or causes inflammation or micro-trauma [4], especially if used frequently, as reported in some regions [5]. Insertion of products into the vagina could also directly cause micro-trauma and/or inflammation. Since the products used are often intended to dry or to tighten the vagina in preparation for sexual intercourse [5], viral entry through breaks in the cervico-vaginal epithelium could be facilitated during or after sex [4]. We found only indirect support in this study for the hypothesis linking intravaginal cleaning with soap, disruption of vaginal flora, and HIV acquisition [12],[20]. Amongst women with normal vaginal flora at baseline, those who reported cleaning with soap were slightly more likely to develop intermediate vaginal flora and BV, possibly because an alkaline pH might promote the growth of BV-associated bacteria. The presence of both intermediate vaginal flora and BV were also associated with an increased incidence of HIV in this study, confirming recent observations [11]. We could not examine the effect of BV in a causal model, as planned. In exploratory analyses, adjustment for BV in addition to demographic and behavioural variables in a standard regression model did not further alter the association between intravaginal cleaning with soap and HIV. The adjusted effect size will not have been estimated precisely in this model, however, because BV is on the hypothesized causal pathway. Contrary to expectation [4], use of household cleaners, vinegar, or lime juice did not increase HIV risk in this study. Lime juice has been reported to cause vaginal epithelial damage in clinical studies [46]. These practices were, however, reported infrequently so our study might have lacked statistical power to answer this question, or measurement error might have reduced our ability to detect modest effects despite pooling data from multiple studies.

### Implications for Research and Policy

It is becoming increasingly important to understand the distribution, motivations for, and health effects of intravaginal practices [12],[47], particularly since a randomised controlled trial has, for the first time, shown that a vaginal microbicide can reduce acquisition of HIV infection [48]. Abdool Karim and colleagues found that the incidence of HIV in women using the antiretroviral agent tenofovir was 39% (95% CI 6%–60%) lower than in women using placebo gel [48]. Whilst reported to be acceptable, there are reasons why intravaginal practices might reduce the effectiveness of microbicides. Women might wash or wipe out microbicides, even when advised not to use habitual intravaginal practices during trials. In Tanzania, about half of women who washed intravaginally reported doing so within 2 h of intercourse [47]. Women who use intravaginal practices might adhere less to vaginal gels, as observed in a trial of the effectiveness of diaphragms and lubricant gel [49]. Alternatively, products inserted into the vagina might react with microbicides, making them inactive or potentially harmful [6]. New female-initiated interventions also need to be developed despite the challenges involved in measuring the impact on preventing HIV acquisition. Behavioural interventions that have been successful in helping young US women to stop vaginal douching [50] might be adapted for women in sub-Saharan Africa to encourage less harmful practices [7] such as use of water alone, which was not associated with an increased risk of HIV acquisition. This study provides evidence to suggest that some intravaginal practices increase the risk of HIV acquisition but a direct causal pathway linking intravaginal cleaning with soap, disruption of vaginal flora, and HIV acquisition has not yet been demonstrated. More consistency is needed in definitions and measurements of intravaginal practices so that the effects of specific intravaginal practices and products can be further elucidated.

## Supporting Information

Table S1 Studies excluded from individual participant data meta-analysis, reasons for exclusion, alphabetical order.
(0.05 MB DOC)

Table S2 Sensitivity analysis for comparison between random and fixed effects models, comparing incidence of HIV in women using intravaginal practices with women using no practice or water only.
(0.03 MB DOC)

Table S3 Sensitivity analysis for associations between intravaginal practices and HIV acquisition, with different reference groups.
(0.03 MB DOC)

Text S1 PRISMA checklist.
(0.13 MB PDF)

## Abbreviations

aHR: adjusted hazard ratio; BV, bacterial vaginosis
CI: confidence interval
HR: hazard ratio
HSV-2: herpes simplex virus infection
IQR: interquartile range
OR: odds ratio; PRISMA, Preferred Reporting Items for Systematic Reviews and Meta-analyses
