# Supplementary material for: Intravaginal Practices, Bacterial Vaginosis, and HIV Infection in Women: Individual Participant Data Meta-analysis
Source: PLoS Med. 2011 Feb 15;8(2):e1000416. doi: 10.1371/journal.pmed.1000416 (PMC3039685; doi:10.1371/journal.pmed.1000416)
Supplement: Table S1 — Studies excluded from individual participant data meta-analysis, reasons for exclusion, alphabetical order. (0.05 MB DOC) [file pmed.1000416.s001.doc]

## Supplementary table 1: Studies excluded from individual patient data meta-analysis, reasons for exclusion, alphabetical order

| Corresponding author or principal Investigator [ref]* | Country | Setting/study population | Enrolled/ followed up, n | Incident HIV, n | Intravaginal practices | Eligible | Reason for exclusion^†^ |
| --- | --- | --- | --- | --- | --- | --- | --- |
| Ghys [26] | Côte d’Ivoire | Sex worker clinic | 542/284 | 26 | Vaginal use of herbs | Not known | Could not contact author |
| Hester [27] | Zambia | Living with HIV positive partner | Not reported/90 | Not known (case-control) | Not known | Not known | Could not contact author |
| Hira [28] | Zambia | Postpartum women | 1720/634 | 19 | ‘Dry sex’ (cloth used to remove vaginal secretions during sex) | Not known | Could not contact author |
| Kapiga [29] | Tanzania | Women working in bars | 845/689 | Not known | Not known | Yes | Data management not complete by deadline |
| Kleinschmidt [30] | South Africa | Women at family planning clinics | 551/551 | 23 | Not measured | No | No data about intravaginal practices |
| Luchters [38] | Kenya | Sex workers | 399/369 | 10 | Vaginal douching or cleaning, drying or tightening | Yes | Agreement not signed by deadline |
| Nagot [31] | Burkina Faso | Sex workers | 279/273 | Not known | Vaginal douching | Not known | Could not contact author |
| Ramjee [40] | South Africa, Tanzania, Zambia | Community level, family planning clinics, bars, hotels | 958/886 | 3.8/100 woman years | Not known | Yes | Declined |
| Rees [39] | South Africa | Family planning clinics | 643/567 | 23 | Vaginal douching, dry sex, cloth to wipe vagina | Yes | Data management not complete by deadline |
| Riedner [32] | Tanzania | Women working in bars | 600/753 | 36 | Not measured | No | No data about intravaginal practices |
| Taha [33] | Malawi | Pregnant and postpartum women | 1169 | 97 | Not measured | Not known | Could not contact author |
| Van de Wijgert [34] | Zimbabwe | Women at family planning, primary care, postnatal clinics | 169 | Not measured | Finger cleansing with substances other than water, wiping inside vagina, inserting traditional substances | No | No data about HIV incidence |

**Legend**:

* [ref] is the reference number of the publication cited in the main manuscript.

† We tried to contact an investigator of all potentially eligible studies, even if the publications did not report a relevant exposure or outcome. For example, three studies in our previous systematic review reported on associations between vaginal infections and incident HIV. We tried to find out whether there were data about intravaginal practices.
