# Supplementary material for: Intravaginal Practices, Bacterial Vaginosis, and HIV Infection in Women: Individual Participant Data Meta-analysis
Source: PLoS Med. 2011 Feb 15;8(2):e1000416. doi: 10.1371/journal.pmed.1000416 (PMC3039685; doi:10.1371/journal.pmed.1000416)
Supplement: Table S2 — Sensitivity analysis for comparison between random and fixed effects models, comparing incidence of HIV in women using intravaginal practices with women using no practice or water only. (0.03 MB DOC) [file pmed.1000416.s002.doc]

## Supplementary table 2: Sensitivity analysis for comparison between random and fixed effects models, comparing incidence of HIV in women using intravaginal practices with women using no practice or water only

| **Intravaginal practice** | **Number in model (strata/studies)** | **Type of model** | **Hazard ratio (95% CI)** | | **Adjusted hazard ratio (95% CI)** | | | | | |
| --- | --- | --- | --- | --- | --- | --- | --- | --- | --- | --- |
|  |  |  | **Unadjusted** | **I^2^ %** | **Demographic/ behavioural factors**^†^ | **I^2^ %** | **Demographic/ behavioural factors + BV^‡^ at baseline** | **I^2^ %** | **Demographic/ behavioural factors + BV^‡^ before seroconversion** | **I^2^ %** |
| Cleaning with soap and water | 11,387 (12/10) | Random effects | 1.20 (0.97, 1.49) | 8.2 | 1.18 (0.94, 1.48) | 14.3 | 1.18 (0.94, 1.49) | 15.8 | 1.18 (0.94, 1.48) | 13.6 |
|  |  | Fixed effects | 1.21 (0.99, 1.48) | 8.2 | 1.19 (0.97, 1.45) | 14.3 | 1.19 (0.98, 1.46) | 15.8 | 1.19 (0.97, 1.45) | 13.6 |
| Cleaning with household products | 7,893 (12/10) | Random effects | 1.20 (0.78, 1.85) | 0.0 | 1.19 (0.77, 1.84) | 0.0 | 1.25 (0.80, 1.94) | 0.0 | 1.18 (0.76, 1.83) | 0.0 |
|  |  | Fixed effects | 1.20 (0.78, 1.85) | 0.0 | 1.19 (0.77, 1.84) | 0.0 | 1.25 (0.80, 1.94) | 0.0 | 1.18 (0.76, 1.83) | 0.0 |
| Cloth to wipe out vagina or apply products | 8,475 (12/10) | Random effects | 1.44 (1.13, 1.82) | 0.0 | 1.38 (1.06, 1.80) | 6.5 | 1.39 (1.06, 1.81) | 6.9 | 1.38 (1.03, 1.85) | 15.9 |
|  |  | Fixed effects | 1.44 (1.13, 1.82) | 0.0 | 1.39 (1.09, 1.77) | 6.5 | 1.40 (1.10, 1.78) | 6.9 | 1.40 (1.10, 1.78) | 15.9 |
| Insertion of products to dry or tighten vagina | 8,216 (11/9) | Random effects | 1.36 (1.01, 1.85) | 0.0 | 1.32 (0.97, 1.79) | 0.0 | 1.33 (0.98, 1.82) | 0.0 | 1.32 (0.97, 1.80) | 0.0 |
|  |  | Fixed effects | 1.36 (1.01, 1.85) | 0.0 | 1.32 (0.97, 1.79) | 0.0 | 1.33 (0.98, 1.82) | 0.0 | 1.32 (0.97, 1.80) | 0.0 |
