# Supplementary material for: Intravaginal Practices, Bacterial Vaginosis, and HIV Infection in Women: Individual Participant Data Meta-analysis
Source: PLoS Med. 2011 Feb 15;8(2):e1000416. doi: 10.1371/journal.pmed.1000416 (PMC3039685; doi:10.1371/journal.pmed.1000416)
Supplement: Table S3 — Sensitivity analysis for associations between intravaginal practices and HIV acquisition, with different reference groups. (0.03 MB DOC) [file pmed.1000416.s003.doc]

## Supplementary table 3: Sensitivity analysis for associations between intravaginal practices and HIV acquisition, with different reference groups

| **Intravaginal practice** | **Unadjusted hazard ratio (95% CI)** | | | | | | | | |
| --- | --- | --- | --- | --- | --- | --- | --- | --- | --- |
|  | **N in model** | **No intravaginal practice or water only** | **I^2^ %** | **N in model** | **Water only** | **I^2^ %** | **N in model** | **No intravaginal practice** | **I^2^ %** |
| **Water only** | .. | .. | .. | .. | .. | .. | 8278 | 1.02 (0.78, 1.35) | 3.9 |
| **Cleaning with soap** | 13071 | 1.25 (1.03, 1.52) | 7.2 | 8520 | 1.29 (1.03, 1.61) | 0.0 | 9047 | 1.16 (0.87, 1.56) | 25.0 |
| **Cleaning with household products** | 8879 | 1.10 (0.73, 1.66) | 0.0 | 4505 | 1.03 (0.67, 1.60) | 0.0 | 4855 | 1.05 (0.62, 1.77) | 0.0 |
| **Use of cloth, paper, tissue** | 10332 | 1.47 (1.19, 1.82) | 0.0 | 6032 | 1.53 (1.16, 2.01) | 0.0 | 7929 | 1.39 (1.04, 1.86) | 4.9 |
| **Insertion of products to dry/tighten** | 9420 | 1.34 (1.03, 1.75) | 0.0 | 5169 | 1.31 (0.98, 1.76) | 0.0 | 5936 | 1.34 (0.95, 1.88) | 0.0 |

**Legend:**

Data from model with no intravaginal practice or water only as the reference group are taken from Figures 1-4
